# Genome-wide identification and characteristic analysis of *ETS* gene family in blood clam *Tegillarca granosa*

**DOI:** 10.1186/s12864-023-09731-5

**Published:** 2023-11-21

**Authors:** Hongyu Jin, Weiwei Zhang, Hongxing Liu, Yongbo Bao

**Affiliations:** 1 School of Marine Sciences, Ningbo University, Ningbo, 315000 China; 2 Zhejiang Key Laboratory of Aquatic Germplasm Resources, College of Biological & Environmental Sciences, Zhejiang Wanli University, Zhejiang, 315100 China

**Keywords:** ETS, Mollusks, Phylogenetic analysis, Expression profiling, Total hemocyte counts

## Abstract

**Background:**

ETS transcription factors, known as the E26 transformation-specific factors, assume a critical role in the regulation of various vital biological processes in animals, including cell differentiation, the cell cycle, and cell apoptosis. However, their characterization in mollusks is currently lacking.

**Results:**

The current study focused on a comprehensive analysis of the ETS genes in blood clam *Tegillarca granosa* and other mollusk genomes. Our phylogenetic analysis revealed the absence of the SPI and ETV subfamilies in mollusks compared to humans. Additionally, several ETS genes in mollusks were found to lack the PNT domain, potentially resulting in a diminished ability of ETS proteins to bind target genes. Interestingly, the bivalve *ETS1* genes exhibited significantly high expression levels during the multicellular proliferation stage and in gill tissues. Furthermore, qRT-PCR results showed that *Tg-ETS-14* (*ETS1*) is upregulated in the high total hemocyte counts (THC) population of *T. granosa*, suggesting it plays a significant role in stimulating hemocyte proliferation.

**Conclusion:**

Our study significantly contributes to the comprehension of the evolutionary aspects concerning the ETS gene family, while also providing valuable insights into its role in fostering hemocyte proliferation across mollusks.

**Supplementary Information:**

The online version contains supplementary material available at 10.1186/s12864-023-09731-5.

## Background

The ETS genes originated from the v-ets gene found in the avian retrovirus E26, which is responsible for causing leukemia. This oncogene was transferred from a homologous gene in the chicken genome and encodes a segment of a hybrid viral protein [1, 2]. Consequently, the human ETS genes (*ETS-1*, *ETS-2*, and *ERG*) and their corresponding proteins were discovered [3]. *ETS* factors have the ability to act as either positive or negative regulators in a range of biological processes, including cellular proliferation, differentiation, invasion, as well as processes such as adhesion, migration, hematopoiesis, and apoptosis [4–6].

The classification of human ETS factors is based on their ETS domain sequence homology, resulting in 12 subgroups: ETS, PEA3, ESE, ETV, TCF, GABP, ELF1, SPI1, TEL, ERF, SPDEF, and ERG [7]. All ETS proteins share a highly conserved ETS domain. This domain is a winged helix-turn-helix structure that interacts with the core DNA sequence 5’-GGA(A/T)-3’ [8]. The third helix primarily determines the DNA-binding specificity. Additionally, around one-third of the ETS family members encode a secondary domain known as the PNT domain, also referred to as the SAM_PNT domain [9]. Furthermore, apart from the two predominant domains mentioned above, the GABP subfamily encompasses a distinct GABPA domain that serves the purpose of enlisting cofactors [10].

The phylogenetic analysis reveals the presence of the ETS gene family across various metazoan phyla, and its expansion from invertebrates to vertebrates likely results from extensive duplications of genomic regions in vertebrates [11–13]. Extensive research has been conducted on the ETS genes in vertebrates, with approximately 30 different types identified in various vertebrate species, including 27 in humans and 26 in mice [2]. Dysregulation of ETS factors in humans leads to the aberrant expression of multiple target genes, which are known to play crucial roles in various processes implicated in cancer progression [14, 15]. In zebrafish, the involvement of ETS gene family members such as *erg*, *fli1*, and *spi2* has been confirmed in angiogenesis and hematopoiesis [16, 17]. In invertebrates, a limited number of ETS genes have been documented. For instance, in *Drosophila melanogaster*, *ELF1*, *Ets1*, *TEL*, and *GABPA* have been observed to function in diverse developmental processes, including metamorphosis, oogenesis, neurogenesis, myogenesis, and eye development [18, 19]. In *Caenorhabditis elegans*, *ETS-5* controls satiety-induced quiescence [20], while *ETS-4* mutations can extend the mean lifespan of adult organisms [21]. However, no instances of ETS genes have been reported in molluscan organisms.

*Tegillarca granosa*, commonly known as the blood clam, is a marine bivalve species that exhibits typical filter-feeding behavior. It is widely distributed across the coastal regions of the Indo-Pacific area. *T. granosa* stands out among invertebrate taxa as for possessing red hemocytes within its hemolymph that are enriched with hemoglobin [22]. The blood color of *T. granosa* is attributed to the abundance of hemoglobin in its hemocytes, resulting in a vibrant red hue [23, 24]. Interestingly, the shade of the blood color is closely associated with the health status and nutritional value of *T. granosa*. A recent study indicated that the changes in blood color shade are influenced, at least in part, by the upregulation and downregulation of genes associated with cell proliferation [24]. The genome sequencing project conducted on *T. granosa* has recently yielded valuable genomic data, enabling systematic analyses of cell proliferation related gene families within this species [25].

Here, we systematically studied the *ETS* genes of eleven species from three classes of mollusks. Through comprehensive analyses of gene structure and spatiotemporal expression patterns, we gained precious understanding of the latent functions of ETS genes in *T. granosa*. Previous research conducted by our team has demonstrated that populations of *T. granosa* with high levels of total hemocyte count (THC) exhibit an increased number of proliferative cells and enhanced hematopoietic potential in comparison to populations with lower levels of THC [24]. Consequently, to expand our comprehension of the hematopoietic function of the ETS genes in *T. granosa*, we conducted further investigation into the transcriptional patterns of various ETS members in high and low THC populations. This is the first comprehensive study of ETS family genes in mollusks, providing useful information for their classification, evolution and function.

The findings of this study serve as a fundamental basis for future investigations into the distribution and biological functions of ETS genes in mollusks and may be beneficial for understanding the hematopoietic mechanism of *T. granosa*.

## Results

### Genome wide identification of *ETS* genes in *T. granosa* and other mollusks

Sixteen ETS genes were identified by searching the translated CDS file of blood clam *T. granosa* (Table 1). The proteins encoded by ETS genes in mollusks have lengths varying from 73 to 1141 amino acids (aa) (Additional file 5). Their isoelectric points (pI) ranged from 4.45 to 10.91. Furthermore, with the exception of Sp-ETS-2 and Sp-ETS-14, the GRAVY value predictions for ETS proteins were negative, indicating that the majority of them exhibited a hydrophilic nature. Except for Bg-ETS-22, other sequences were not predicted to contain signal peptides. Subcellular localization analysis revealed that the majority of ETS proteins were predominantly localized to the nucleus, with a small fraction also distributed in the cytoplasm, mitochondria, Golgi apparatus, and extracellular space.

**Table 1:** Classification of ETS genes from eleven mollusks

Subfamily	Type	*Tegillarca granosa*	*Crassostrea gigas*	*Mizuhopecten yessoensis*	*Mytilus galloprovincialis*	*Sinonovacula constricta*	*Octopus bimaculoides*	*Octopus sinensis*	*Sepia pharaonis*	*Aplysia californica*	*Biomphalaria glabrata*	*Lottia gigantea*
**ERF**	**ERF**	0	0	0	0	0	1	3	0	0	0	0
	**ETV3L**	0	0	0	0	0	0	2	0	0	0	0
**ERG**	**Fli1**	1	1	3	2	0	0	0	0	0	0	0
	**ERG**	1	1	0	0	0	0	0	0	0	0	0
	**FEV**	2	6	4	4	3	3	3	1	4	3	0
**TCF**	**ELK1**	1	4	3	1	1	0	0	0	1	0	0
	**ELK3**	0	0	0	2	0	0	3	1	0	9	0
**PEA3**	**ETV1**	1	2	1	2	0	0	0	1	0	0	1
	**ETV4**	0	0	0	0	1	0	0	0	1	0	0
**GABP**	**GABPA**	1	2	2	1	1	1	2	1	3	0	1
**ETS**	**ETS1**	1	5	5	2	0	3	9	2	1	1	0
**ESE**	**EHF**	2	12	7	4	2	1	3	3	1	1	0
	**ELF5**	0	0	0	0	0	0	0	0	0	0	1
**TEL**	**ETV6**	0	18	7	7	0	1	3	0	0	3	0
	**ETV7**	0	0	0	0	0	0	0	1	0	0	0
**ELF**	**ELF2**	3	8	9	5	1	1	3	1	3	1	1
**PDEF**	**SPDEF**	1	4	1	2	1	0	1	4	2	2	0
**No classification**		2	3	2	14	2	4	3	2	5	2	6
**Total**		16	66	44	46	12	15	35	17	21	22	10

### Phylogenetic analysis

**Fig. 1:**
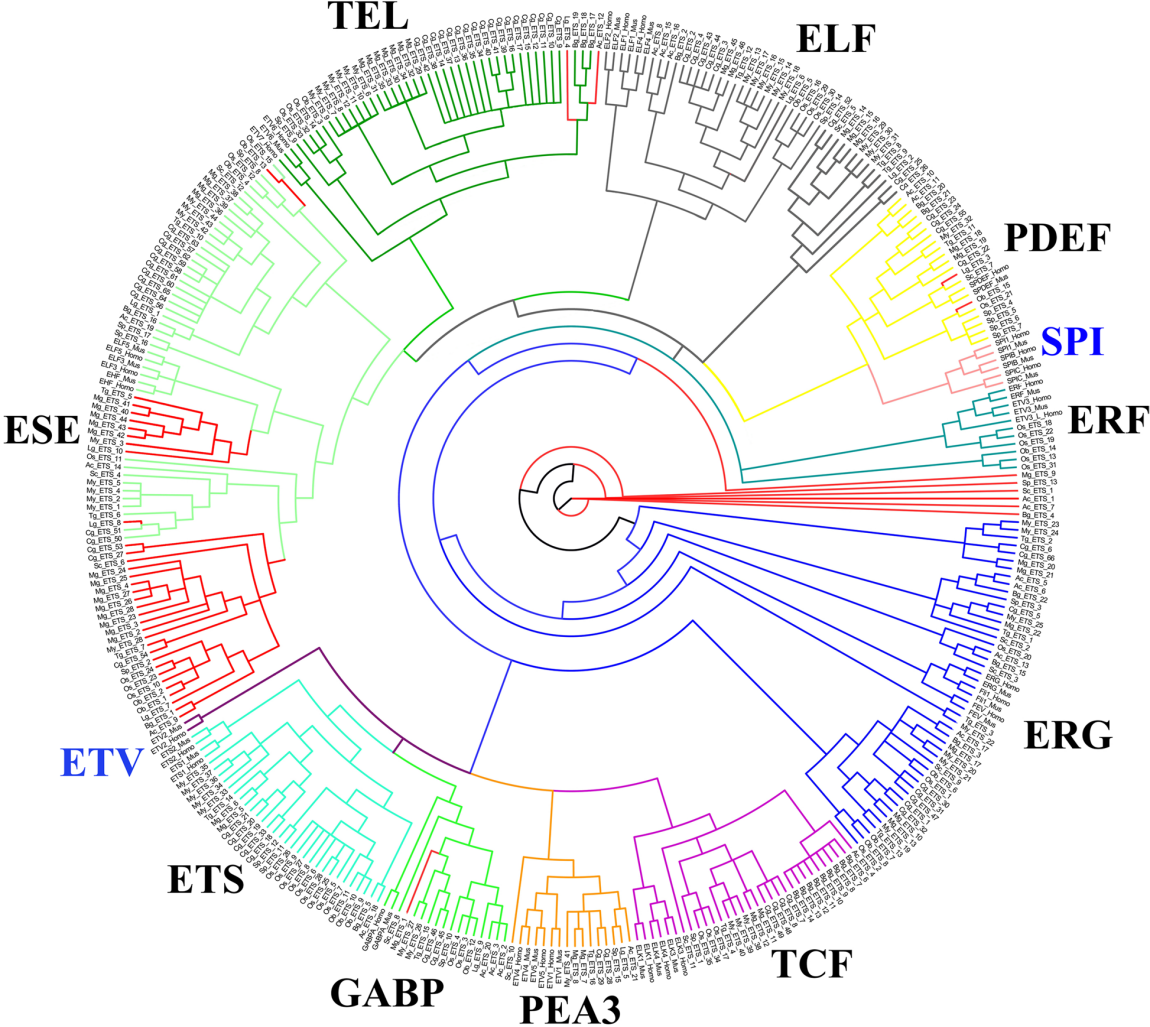
Phylogenetic analysis of ETS proteins of human, mice and eleven mollusks using BI analysis. Different branch colors indicate different subfamilies, and red branches represent unclassified families due to domain missing or no matching similar reference sequences. The blue fonts represent the absence of the subfamily in mollusks

The phylogenetic analysis revealed the subdivision of all ETS proteins in mollusks into ten distinct subfamilies. Compared to humans and mice, SPI and ETV subfamily genes were absent in mollusks (Fig. 1). The ERF subfamily genes were identified in only two species of mollusks, namely *O. bimaculoides* and *O. sinensis*. In mollusks, the ERG subfamily, consisting of 42 gene sequences, is the largest subfamily, while the ERF subfamily, consisting of only 6 gene sequences, is the smallest subfamily.

### Gene structure, conserved motif and protein tertiary structure

**Fig. 2:**
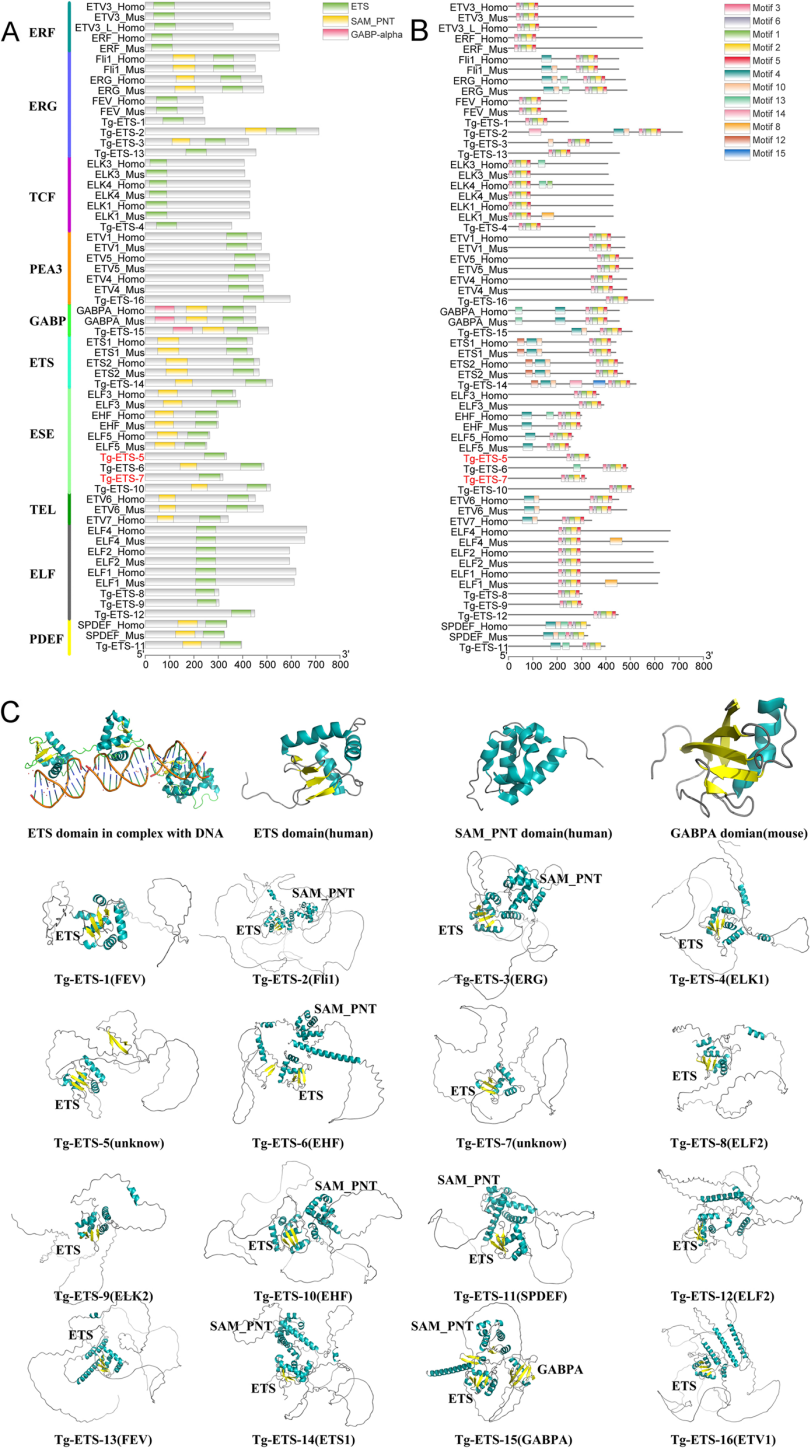
Domain (**A**) and motif (**B**) architecture analysis of *ETS* genes in *T. granosa* and reference genes in *H. sapiens* and *M. musculus*. The genes highlighted in red are deficient in the SAM_PNT domain. For more information on other ETS gene domains and motif, please refer to Additional file 1. **C** The ETS protein tertiary structure of *T. granosa* was predicted by AlphaFold2 and the best result was selected based on software recommendations

Predictions were made for the conserved domain and motif of ETS proteins. (Fig. 2A and B). It is noteworthy that certain sequences of mollusk ETS (Ac-ETS-9, Bg-ETS-1, Cg-ETS-27/53/54, Lg-ETS-7/8/10, Mg-ETS-2/3/23/24/25/26/27/28/40/41/42/43/44, My-ETS-3/8, Ob-ETS-1/2/13, Os-ETS-10/23/24, Sc-ETS-6, Sp-ETS-2, Tg-ETS-5/7) were observed to cluster together with the ESE subfamily in constructed phylogenetic trees but lack the PNT domain (Additional file 1). Moreover, the TEL subfamily encompasses Ac-ETS-12 and Lg-ETS-4, the PDEF subfamily includes Ob-ETS-15 and Lg-ETS-3, and the ELF subfamily comprises Lg-ETS-6, all of which underwent a loss of the PNT domain (Additional file 1). In addition, the ETS protein sequences of *T. granosa* were selected for three-dimensional (3D) structure prediction (Fig. 2C) and annotated the structural domains. Figure 2C illustrates the structural composition of the ETS domain in blood clam *T. granosa*, which includes three alpha-helices and either four or three anti-parallel beta-sheets. Additionally, the PNT domain is composed of four alpha-helices and one small alpha-helix. Moreover, the GABPA domain consists of four anti-parallel beta-sheets and one small alpha-helix. Moreover, by comparing the ETS, PNT and GABPA domain of *H. sapiens* and *M. musculus*, the ETS proteins of *T. granosa* also share similar 3D structure, indicating a potential functional similarity between the ETS proteins of *T. granosa* and those of *H. sapiens* and *M. musculus*.

### Chromosomal locations

**Fig. 3:**
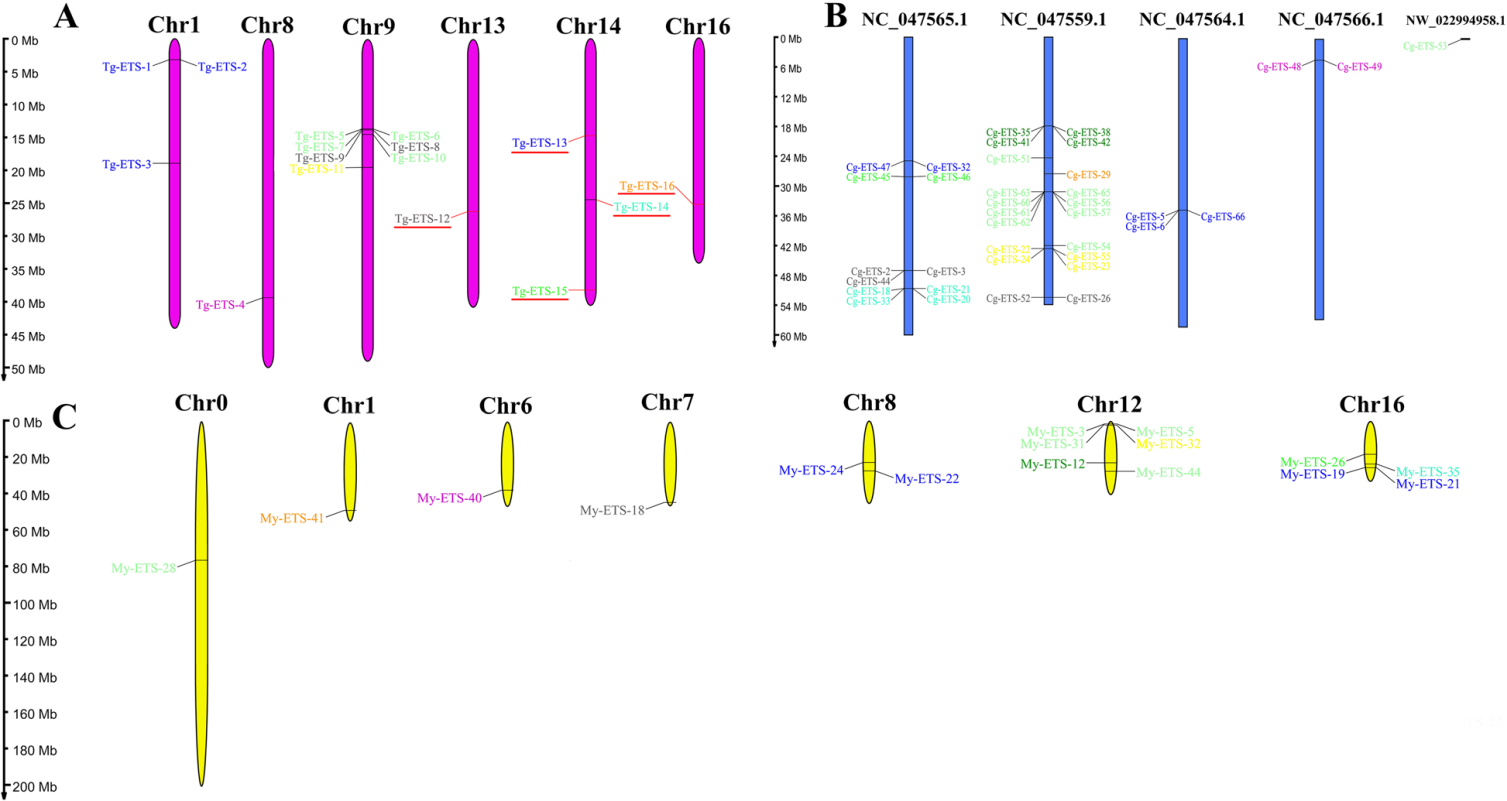
Chromosome distribution of ETS genes in three bivalve *T. granosa* (**A**), *C. gigas* (**B**), and *M. yessoensis* (**C**). For the sake of conciseness, ETS proteins produced by different splicing modes of the same gene in C. gigas and *M. yessoensis* were hidden. The color of each gene indicated the corresponding family, and the assigned color scheme was congruent with that presented in Fig. 1. The present study highlighted the *ETS* genes that exhibit statistically significant differences in expression levels between the high and low THC of *T. granosa*, as indicated by the genes that were denoted by the red underline

The genomic locations of the 16 genes of *T. granosa* were determined to be on six chromosomes, while the 66 genes of *C. gigas* were mapped to four chromosomes and one contig. Moreover, the genomic locations of the 44 genes of *M. yessoensis* were found to be distributed in seven chromosomes (Fig. 3). The ERG subfamily genes tend to be distributed on two chromosomes. In *T. granosa*, the ERG subfamily genes were found to be situated on chromosomes 1 and 14. Likewise, in *C. gigas*, these genes were positioned on chromosomes NC_047565.1 and NC_047564.1, whereas in *M. yessoensis*, they were located on chromosomes 8 and 16. In addition, other subfamily genes tend to be distributed on the same chromosome.

The findings revealed that in *T. granosa*, two chromosomes contained three unevenly distributed tandem repeat gene pairs. In *C. gigas*, four chromosomes harbored nine tandem duplicate gene pairs that were also unevenly distributed. Furthermore, in *M. yessoensis*, two chromosomes contained two unevenly distributed tandem duplicate gene pairs (Additional file 6). In *T. granosa*, each of the ERG, ESE, and ELF subfamilies had one tandem repeat pair. In *C. gigas*, the ERG and ELF subfamilies had two tandem repeat pairs, while the ESE, ETS, GABPA, TEL, and TCF subfamilies each had one tandem repeat pair. In *M. yessoensis*, the ERG and ESE subfamily had one tandem repeat pairs.

### Expression analysis

**Fig. 4:**
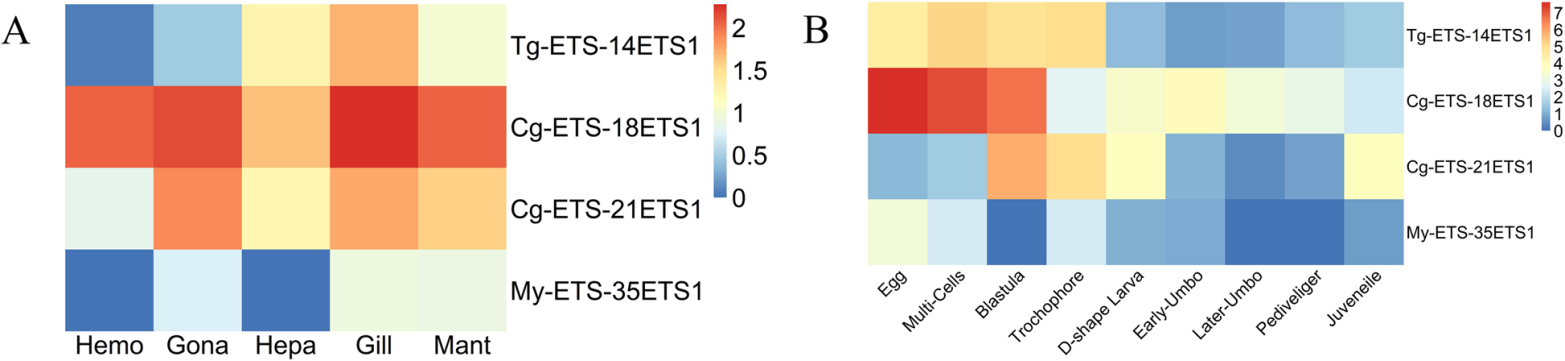
Heatmap of *ETS1* expression profiles (TPM) in different developmental stages and different tissues in three bivalves. The heatmap utilizes a blue-to-red color scale to depict the relative expression levels, where blue indicates low expression, and red represents high expression. **A** Expression of *ETS1* genes during embryonic development. **B** Expression of *ETS1* genes in adult tissues. For more information on other ETS gene expression profiles, please refer to Additional file 2

To gain a deeper understanding of the characteristics and functions of ETS genes in mollusks, comprehensive transcriptome resources were employed to analyze and assess the temporal and spatial expression patterns of ETS genes in three bivalve mollusks (Additional file 7). Our research demonstrated that approximately half of the *ETS* genes exhibited low or no transcriptional expression in different developmental stages and adult tissues, and these genes with low expression levels were mainly distributed in *C. gigas* and *M. yessoensis*. Furthermore, during different developmental stages, *ETS1* (*Tg-ETS-14*, *Cg-ETS-18/21*, *My-ETS-35*) of *T. granosa*, *C. gigas* and *M. yessoensis* were highly expressed in the process of multicellular cleavage, and their transcription levels gradually or rapidly decreased from the trochophore (Fig. 4A). Meanwhile, in different adult tissues, *ETS1* genes of *M. yessoensis, C. gigas* and *T. granosa* were highly expressed in the gill (Fig. 4B).

### qRT-PCR analysis in high and low THC populations of* T. granosa*

**Fig. 5:**
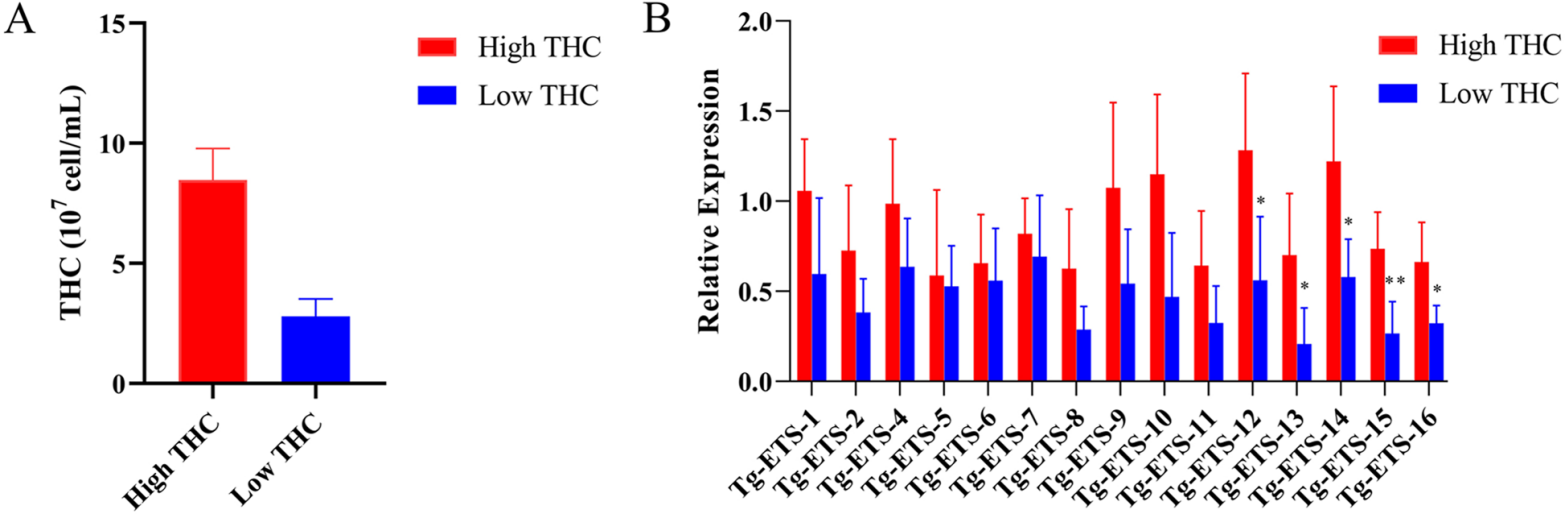
**A** The total hemocyte count (THC) of *T. granosa* high and low THC populations. For more information on THC of *T. granosa*, please refer to Additional file 3. **B** Relative *ETS* genes expression in gill of *T. granosa* high and low THC populations

To explore the potential biological functions of the ETS gene family in *T. granosa*, the mRNA expression levels of ETS genes were assessed in populations with high and low THC (Fig. 5B). The findings demonstrated that, in comparison to the low THC population, the mRNA expression of the *GABPA* (*Tg-ETS-15*) gene exhibited a significant upregulation in the high THC *T. granosa* population. Additionally, the ELF2 (Tg-ETS-12), FEV (Tg-ETS-13), ETS1 (Tg-ETS-14), and ETV1 (Tg-ETS-16) genes also displayed significant upregulation.

## Discussions

ETS transcription factors are known to exert a pivotal influence on diverse fundamental biological processes in animals, encompassing cell differentiation, cell cycle control, cellular apoptosis, and various other indispensable regulatory pathways. Nevertheless, the comprehensive understanding of their functional attributes within mollusks remains notably deficient. This study focused on identifying and characterizing the ETS family in eleven mollusks using genomic and transcriptomic data. Through subcellular localization prediction analysis, it was discovered that the majority of ETS sequences are localized in the nucleus. This observation suggests a potential correlation between their localization and their role in transcriptional regulation [26, 27].

According to the phylogenetic analysis, mollusks have only 17 types of *ETS* genes belonging to 10 subfamilies, which is fewer than the number found in vertebrates such as humans and mice, with 28 and 27 types of ETS genes respectively [8].The widespread presence of low-complexity ETS binding sites suggests the potential existence of functional redundancy among ETS factors [28]. As a result, gene loss in the mollusk ETS family may be attributed to this redundancy. Furthermore, it is noteworthy that genes from the SPI and ETV subfamilies have not been identified in mollusks. The SPI subfamily genes are known to play roles in B cell development [29, 30] and the maintenance of homeostasis in Red pulp macrophages [31]. *ETV2* (The only gene in the ETV subfamily) mutant embryos had exhibited deficiencies in endocardial lineages and had demonstrated notable vessel malformations [32–34]. It is important to note that mollusks do not possess B cells or Red pulp macrophages, which are specific to vertebrates and absent in mollusks [35–37], nor do they possess a sophisticated cardiovascular system [38], which may be one contributing factor to the absence of SPI and ETS subfamily genes. Notably, the ERF and *ETV3L* genes of the ERF subfamily are only found in *Octopus*. In *Xenopus laevis*, loss-of-function analysis revealed that *ERF* and *ETV3L* play a crucial role in inhibiting the proliferation of neural progenitors, thereby facilitating their differentiation. Conversely, overexpression of *ERF* led to an increase in the number of primary neurons [39]. Meanwhile, *Octopuses* are one of the most intelligent invertebrates. Their sensory and motor systems are highly developed, and they possess a large, multilobed brain that facilitates complex analysis, learning, and behavioral control [40]. Therefore, we speculate that the complex nervous system in *Octopuses* might be the reason for the presence of the ERF subfamily genes, compared to other mollusks. It is plausible that the *ERF* and *ETV3L* genes could potentially play a significant role in the differentiation of *Octopus* nerve cells.

In our study, we had discovered a frequent absence of the PNT domain within ETS genes of mollusks, with the most striking occurrences observed within the ESE subfamily (including *Tg-ETS-5* and *Tg-ETS-7* in *T. granosa*) (Fig. 2). However, the absence of PNT domain was also observed in certain human ETS sequences [26]. In-depth in vitro kinetic studies have provided evidence that the PNT domain functions by facilitating signal transduction through enhanced substrate binding. This enhancement occurs in interactions such as *ETS1* or *ETS2* with MAP kinase ERK2 and *Drosophila PNT-p2* with normal roll kinase [41–43]. Therefore, the PNT domains act as docking modules, engaging with corresponding docking sites found on the kinases, rather than directly altering the enzymatic kinetics [44, 45]. Presumably, this boosts the specificity and modification rate of adjacent phosphoacceptors at enzyme catalytic sites by effectively increasing their local concentration [46]. Hence, it seems reasonable that nearly half of the members in the ESE subfamily of invertebrates lack the PNT domain. These genes, which lack the PNT domain, may have weaker binding capacity compared to the normal ETS genes in mollusks.

In vertebrates, ETS1 plays a crucial role in cell proliferation, differentiation, and vascular development [47–49]. Our findings revealed that the expression of bivalve *ETS1* genes is notably higher in gill tissues compared to other tissues. Given that the gill is considered the primary hematopoietic organ in bivalves [24, 50], this suggests that *ETS1* genes in the three bivalves may be involved in regulating hemocyte proliferation and differentiation. Additionally, our observations indicate that bivalve *ETS1* genes exhibit elevated mRNA expression levels during the multicellular proliferation stage, with transcription levels decreasing rapidly from the trochophore stage. This implies the involvement of *ETS1* genes in the regulation of cell proliferation. Furthermore, additional experiments demonstrated a significant increase in the transcriptional expression levels of the *ETS1* gene in the high THC populations of *T. granosa* compared to those with low THC. This suggests that the *ETS1* gene plays an important role in promoting hemocyte proliferation in *T. granosa*.

## Conclusion

In this study, sixteen *ETS* gene sequences belonging to nine subfamilies (ERG, TCF, PEA3, GABPA, ETS, ESE, TEL and ELF) were identified from *T. granosa*. We had observed the absence of PNT domain in the ETS gene family of mollusks, particularly evident in the ESE subfamily. The loss of the PNT domain could lead to a reduced capacity of ETS proteins to effectively bind target genes. Nonetheless, further biochemical and biological investigations are required to ascertain and validate the functional distinctions of these genes lacking PNT domain from other genes in ETS family. The examination of expression profiles during embryonic development and in adult organs provides valuable insights into the function of bivalve ETS genes. The expression patterns of *ETS1* in different developmental stages and adult tissues of three bivalve species, as well as the significantly higher mRNA expression levels in high THC populations compared to low THC populations in *T. granosa*, suggest an important role for ETS1 in hemocyte proliferation in *T. granosa*. This study presents a comprehensive genome-wide characterization of the ETS gene family in *T. granosa* and other mollusks. The findings contribute to a deeper understanding of the function and evolution of the ETS family in mollusks.

## Materials and methods

### Genome-wide identification of ETS genes in *T. granosa* and other mollusks

The genome data of *Mytilus galloprovincialis*, *C. gigas*, *M. yessoensis*, *Sinonovacula constricta*, *Octopus bimaculoides*, *Sepia pharaonis*, *Octopus sinensis*, *Aplysia californica*, *Lottia gigantea*, *Biomphalaria glabrata* were obtained from the National Center for Biotechnology Information (NCBI) database, accessible at https://www.ncbi.nlm.nih.gov/ (last accessed on July 15, 2022). *T. granosa* genome sequences employed in this study were derived from data submitted by our laboratory (BioProject accession PRJNA593692).

To screen the genome data of eleven species for ETS genes, we utilized HMMsearch v3.3.2. The ETS.hmm profile was acquired from PFAM (http://pfam.xfam.org/, accession PF00178; last accessed July 15, 2022). SMART (https://smart.embl.de/; last accessed July 15, 2022), PFAM, and CDSearch (https://www.ncbi.nlm.nih.gov/Structure/cdd/wrpsb.cgi; last accessed July 15, 2022) were utilized to confirm the ETS domains in ETS genes.

### Phylogenetic analysis

Known ETS protein sequences from Human (*Homo sapiens*) and Mice (*Mus musculus*) were downloaded from NCBI. The E-INS-I strategy was used to perform multiple sequence alignment of the amino acid sequences of ETS genes from 11 mollusks and reference ETS sequences from humans and mice, using MAFFT v7.505. The phylogenetic analysis using Bayesian inference was performed with MrBayes v3.2.8. Four Markov chains were employed for 600,000 generations, and sampling was carried out every 2,000 generations. To obtain consensus trees and posterior probability distribution, the initial 25% of trees were excluded. Subsequently, FigTree v1.4.3 was utilized to further analyze the Bayesian trees. BLAST v2.13.0 was utilized to identify the gene types to which sequences belonging to the same subfamily belong.

### Gene structure, conserved motif and protein tertiary structure

MEME (https://meme-suite.org/meme/; accessed on July 15, 2022) was utilized to conduct the analysis of conserved motifs, with motif number set to 15. Subsequently, all the findings from the gene structure and motif analysis were imported into TBtools [51]. AlphaFold2 was employed for predicting the tertiary structure of proteins [52], and PyMOL v2.5.3 was used for visualizing the protein tertiary structure [53].

### Chromosomal locations

ETS genes were mapped to chromosomes based on the chromosomal position provided in the *T. granosa*, *C. gigas* and *M. yessoensis* genome database. A distribution map of ETS genes was protracted using TBtools [51]. The identification method of Gene duplication is referred to this paper [54]. Two genes located in the same chromosomal fragment of less than 100 kb and separated by five or fewer genes were identified as tandem duplicate genes [55].

### Expression analysis

The TPM (transcripts per kilobase of exon model per million mapped reads) were summarized from the published RNA-seq datasets of *T. granosa* [25], *C. gigas* [56] and *M. yessoensis* [57]. During development, eleven embryo/larval developmental stages were chosen to perform expression analysis, including egg; multi-cells; blastula; trochophore; D-shaped larvae; early-umbo larvae and later-umbo larvae; pediveliger; and juvenile. For adults, five tissues (hemolymph, gonad, hepatopancreas, gill, and mantle) were chosen to perform expression analysis. RNASeq reads were cleaned with fastp v0.23.2 [58]. RNASeq reads were mapped to reference genome using RSEM v1.3.3 [59] and STAR v2.4.0j [60] to estimate gene expression. The expressional heatmaps were displayed by the R package Clusterprofiler [61]. Genes with an expression level less than 1 (TPM) across all tissues or developmental stages will be hidden.

### qRT-PCR analysis in high and low THC populations of *T. granosa*

About 600 blood clams were sampled from the same pond from a blood clam farmer in Ningbo City, Zhejiang Province, China. Under controlled conditions of temperature (27.3 ± 0.48 °C) and salinity (26.18 ± 0.64‰), the blood clams were cultured in filtered seawater. They were fed with *Chlorella vulgaris*, with a concentration of 1 mL of concentrated algal solution (20,000 cells/mL) per liter of seawater. The feeding occurred twice daily, at 8:30 AM and 8:30 PM. After a week of continuous cultivation, healthy clams (showing normal blood color and distinct stress response) were chosen for further experiments.

Fifty blood clams from the same growth environment were selected for counting blood cells by microscope (Nikon eclipse E100) (Additional file 3). The *T. granosa* individuals were arranged in descending order based on THC levels, with the top 10% (THC > 7 × 10^7^ cell/ml) considered as the high THC group, and the bottom 10% (THC < 3.4 × 10^7^ cell/ml) regarded as the low THC group (Fig. 5A). The blood clam with the top 10 percent and the bottom 10 percent of THC were selected for RNA extraction from gill. The method of gill tissue RNA extraction and qRT-PCR is consistent with a previous article [24]. The specific primers listed in Additional file 4 were used for qRT-PCR. Data were processed with R package Stats, and significance between the groups was calculated by t-test.

### Supplementary information

**Additional file 1.** Domain and motif architecture analysis of ETS genes in mollusks.

**Additional file 2.** Heatmap of mRNA expression levels of ETS genes in different developmental stages and adult tissues.

**Additional file 3.** Total hemocyte counts (THC) of fifty blood clams from the same growth environment.

**Additional file 4.** Primers used for qRT-PCR.

**Additional file 5. **Genome wide identification of ETS genes in eleven mollusks.

**Additional file 6.** Tandem repeat gene pairs of three bivalve mollusks.

**Additional file 7.** Expressions of ETS genes in different developmental stages and adult tissues.

## Data Availability

The data underlying this article are available in the article and in its online Additional files.
