# Supplementary figures and images for: Genome-wide identification and characteristic analysis of ETS gene family in blood clam Tegillarca granosa
Source: BMC Genomics. 2023 Nov 21;24:700. doi: 10.1186/s12864-023-09731-5 (PMC10664356; doi:10.1186/s12864-023-09731-5)

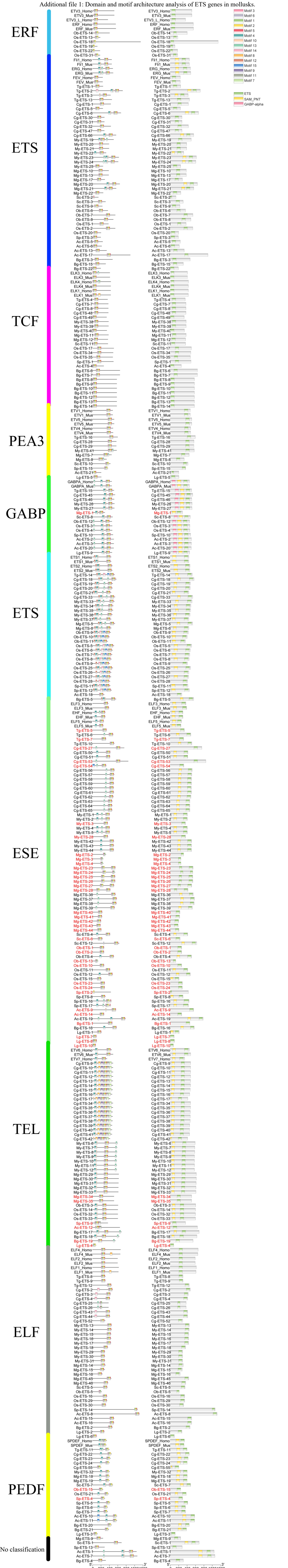

Supplement: Supplementary file 1 — Additional file 1. Domain and motif architecture analysis of ETS genes in mollusks. [file 12864_2023_9731_MOESM1_ESM.pdf]
