# Supplementary material for: Genome-wide identification and characteristic analysis of ETS gene family in blood clam Tegillarca granosa
Source: BMC Genomics. 2023 Nov 21;24:700. doi: 10.1186/s12864-023-09731-5 (PMC10664356; doi:10.1186/s12864-023-09731-5)

Additional file 2: Heatmap of mRNA expression levels of ETS genes in different developmental stages and adult tissues.

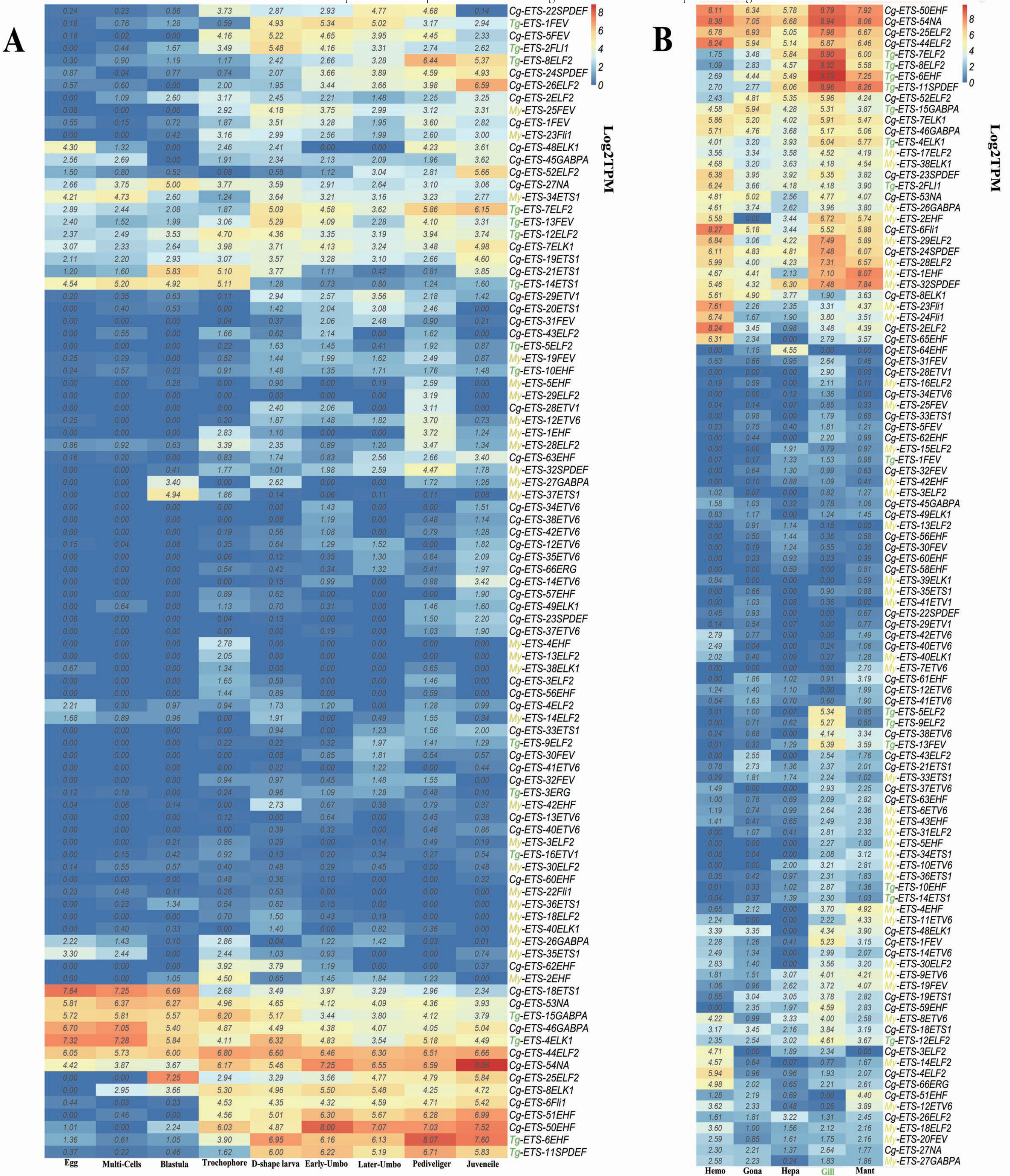

Supplement: Supplementary file 2 — Additional file 2. Heatmap of mRNA expression levels of ETS genes in different developmental stages and adult tissues. [file 12864_2023_9731_MOESM2_ESM.pdf]
