# Supplementary material for: Genome-wide identification and characteristic analysis of ETS gene family in blood clam Tegillarca granosa
Source: BMC Genomics. 2023 Nov 21;24:700. doi: 10.1186/s12864-023-09731-5 (PMC10664356; doi:10.1186/s12864-023-09731-5)
Supplement: Supplementary file 3 — Additional file 3. Total hemocyte counts (THC) of fifty blood clams from the same growth environment. [file 12864_2023_9731_MOESM3_ESM.docx]

**Additional file 3:** Total hemocyte counts (THC) of fifty blood clams from the same growth environment

| Number | THC(107cell/ml) | Number | THC(107cell/ml) |
| --- | --- | --- | --- |
| 1 | 5.6 | 26 | 4.2 |
| 2 | 5.3 | 27 | 6.9 |
| 3 | 5.7 | 28 | 5.4 |
| 4 | 7 | 29 | 4.9 |
| 5 | 6.9 | 30 | 9.1 |
| 6 | 4.6 | 31 | 5.4 |
| 7 | 7.4 | 32 | 4.8 |
| 8 | 5.1 | 33 | 5 |
| 9 | 2.5 | 34 | 4.4 |
| 10 | 10 | 35 | 4.5 |
| 11 | 4.7 | 36 | 4.7 |
| 12 | 6.4 | 37 | 5.1 |
| 13 | 4.5 | 38 | 5.4 |
| 14 | 5.1 | 39 | 6.3 |
| 15 | 3.6 | 40 | 6.9 |
| 16 | 5.8 | 41 | 4.8 |
| 17 | 3 | 42 | 5.3 |
| 18 | 4.5 | 43 | 6.8 |
| 19 | 3.4 | 44 | 5.1 |
| 20 | 7.4 | 45 | 4.5 |
| 21 | 4.7 | 46 | 3.8 |
| 22 | 4.5 | 47 | 3.5 |
| 23 | 6.7 | 48 | 3.4 |
| 24 | 4.2 | 49 | 6.9 |
| 25 | 1.7 | 50 | 5.3 |
