# Supplementary material for: Genome-wide identification and characteristic analysis of ETS gene family in blood clam Tegillarca granosa
Source: BMC Genomics. 2023 Nov 21;24:700. doi: 10.1186/s12864-023-09731-5 (PMC10664356; doi:10.1186/s12864-023-09731-5)
Supplement: Supplementary file 4 — Additional file 4. Primers used for qRT-PCR. [file 12864_2023_9731_MOESM4_ESM.docx]

**Additional file 4:** Primers used for qRT-PCR

| Primer name | Primer sequence |
| --- | --- |
| Tg-ETS-1 F | 5' AGCAGCCAGACTATTACCG 3' |
| Tg-ETS-1 R | 5' GGATATGCTGGACTTTGTG 3' |
| Tg-ETS-2 F | 5' AACGGAAGAGCAAGCCAAAT 3' |
| Tg-ETS-2 R | 5' CCTGTGAAAGCCCTACGAAAT 3' |
| Tg-ETS-4 F | 5' ATTTCCTCACTTTGCCTCA 3' |
| Tg-ETS-4 R | 5' ATCCTCTGTTCTTGGTGGC 3' |
| Tg-ETS-5 F | 5' AACTAGCGAAATAGCCCGTAT 3' |
| Tg-ETS-5 R | 5' CCAAGAACAGGTGCCAAAA 3' |
| Tg-ETS-6 F | 5'CATTGGTGGAACTTGGTAC3' |
| Tg-ETS-6 R | 5'CATTGCCTAATTCACTGTTT3' |
| Tg-ETS-7 F | 5' AGGCTCTACAGGTGATGGC 3' |
| Tg-ETS-7 R | 5' GGTAACAATGGTTCGGATT 3' |
| Tg-ETS-8 F | 5' CAACTTCTTAAAGGACCAG 3' |
| Tg-ETS-8 R | 5' GTAGTAGTGCCTTATAGCC 3' |
| Tg-ETS-9 F | 5' TGCCTTGCTTTGACGAACT 3' |
| Tg-ETS-9 R | 5' CAGCGAACCAAATCTGGACTG 3' |
| Tg-ETS-10 F | 5' AAAAGGGACGTTAAATCGG 3' |
| Tg-ETS-10 R | 5' TTGGCAAAGTTACCACAGC 3' |
| Tg-ETS-11 F | 5' ACAGTAGCTCCAGCTCATC 3' |
| Tg-ETS-11 R | 5' TTATGTTCGCTTTCACCAG 3' |
| Tg-ETS-12 F | 5' TTACAACCGCCACCAGCAC 3' |
| Tg-ETS-12 R | 5' TGGGCGAATGACAACCTGA 3' |
| Tg-ETS-13 F | 5' GGATTCGCCCGAGTGTAGT 3' |
| Tg-ETS-13 R | 5' TGCTTTGGGTCTTGGTTCT 3' |
| Tg-ETS-14 F | 5' CAGCCTTATCAGATGGTTC 3' |
| Tg-ETS-14 R | 5' TCAGTATGTGGCGGGTATT 3' |
| Tg-ETS-15 F | 5' AGTCGTCATCCCACTACCG 3' |
| Tg-ETS-15 R | 5' CTCCTTTATGGCCCATTCT 3' |
| Tg-ETS-16 F | 5' AAGGCATGGAAATGAAAGC 3' |
| Tg-ETS-16 R | 5' CCACAACTGTAATGAACCC 3' |
