# Supplementary material for: Genome-wide identification and characteristic analysis of ETS gene family in blood clam Tegillarca granosa
Source: BMC Genomics. 2023 Nov 21;24:700. doi: 10.1186/s12864-023-09731-5 (PMC10664356; doi:10.1186/s12864-023-09731-5)
Supplement: Supplementary file 6 — Additional file 6. Tandem repeat gene pairs of three bivalve mollusks. [file 12864_2023_9731_MOESM6_ESM.docx]

**Additional file 6:** Tandem repeat gene pairs of three bivalve mollusks

| Gene Name | Gene ID | Gene Name | Gene ID | Duplication Type | Location |
| --- | --- | --- | --- | --- | --- |
| Tg-ETS-1 | Pec0131920.1 | Tg-ETS-2 | Pec0131930.1 | Tandem duplication | Chr1 |
| Tg-ETS-5 | Pec0131930.1 | Tg-ETS-6 | Pec0109280.1 | Tandem duplication | Chr9 |
| Tg-ETS-6 | Pec0109280.1 | Tg-ETS-7 | Pec0109290.1 | Tandem duplication | Chr9 |
| Tg-ETS-8 | Pec0109300.1 | Tg-ETS-9 | Pec0109320.1 | Tandem duplication | Chr9 |
| Cg-ETS-47 | XP_034303636.1 | Cg-ETS-32 | XP_019919757.2 | Tandem duplication | NC_047565.1 |
| Cg-ETS-45 | XP_034302152.1 | Cg-ETS-46 | XP_034302153.1 | Tandem duplication | NC_047565.1 |
| Cg-ETS-2 | XP_011417409.2 | Cg-ETS-3 | XP_011417410.2 | Tandem duplication | NC_047565.1 |
| Cg-ETS-3 | XP_011417410.2 | Cg-ETS-44 | XP_034300886.1 | Tandem duplication | NC_047565.1 |
| Cg-ETS-19 | XP_011436350.2 | Cg-ETS-20 | XP_011436351.1 | Tandem duplication | NC_047565.1 |
| Cg-ETS-20 | XP_011436351.1 | Cg-ETS-21 | XP_011436352.1 | Tandem duplication | NC_047565.1 |
| Cg-ETS-21 | XP_011436352.1 | Cg-ETS-33 | XP_019925424.1 | Tandem duplication | NC_047565.1 |
| Cg-ETS-35 | XP_034300690.1 | Cg-ETS-38 | XP_034300721.1 | Tandem duplication | NC_047559.1 |
| Cg-ETS-38 | XP_034300721.1 | Cg-ETS-41 | XP_034300740.1 | Tandem duplication | NC_047559.1 |
| Cg-ETS-41 | XP_034300740.1 | Cg-ETS-42 | XP_034300750.1 | Tandem duplication | NC_047559.1 |
| Cg-ETS-56 | XP_034325844.1 | Cg-ETS-57 | XP_034325852.1 | Tandem duplication | NC_047559.1 |
| Cg-ETS-57 | XP_034325852.1 | Cg-ETS-60 | XP_034325872.1 | Tandem duplication | NC_047559.1 |
| Cg-ETS-60 | XP_034325872.1 | Cg-ETS-61 | XP_034325880.1 | Tandem duplication | NC_047559.1 |
| Cg-ETS-61 | XP_034325880.1 | Cg-ETS-62 | XP_034325888.1 | Tandem duplication | NC_047559.1 |
| Cg-ETS-62 | XP_034325888.1 | Cg-ETS-63 | XP_034325897.1 | Tandem duplication | NC_047559.1 |
| Cg-ETS-63 | XP_034325897.1 | Cg-ETS-65 | XP_034325911.1 | Tandem duplication | NC_047559.1 |
| Cg-ETS-22 | XP_011443809.2 | Cg-ETS-23 | XP_011443810.2 | Tandem duplication | NC_047559.1 |
| Cg-ETS-23 | XP_011443810.2 | Cg-ETS-24 | XP_011443811.2 | Tandem duplication | NC_047559.1 |
| Cg-ETS-24 | XP_011443811.2 | Cg-ETS-55 | XP_034320659.1 | Tandem duplication | NC_047559.1 |
| Cg-ETS-26 | XP_011447492.2 | Cg-ETS-52 | XP_034317332.1 | Tandem duplication | NC_047559.1 |
| Cg-ETS-5 | XP_011419494.1 | Cg-ETS-6 | XP_011420416.2 | Tandem duplication | NC_047564.1 |
| Cg-ETS-6 | XP_011420416.2 | Cg-ETS-66 | XP_034335820.1 | Tandem duplication | NC_047564.1 |
| Cg-ETS-48 | XP_034307000.1 | Cg-ETS-49 | XP_034307001.1 | Tandem duplication | NC_047566.1 |
| My-ETS-3 | XP_021347935.1 | My-ETS-5 | XP_021347940.1 | Tandem duplication | Chr12 |
| My-ETS-5 | XP_021347940.1 | My-ETS-31 | XP_021363796.1 | Tandem duplication | Chr12 |
| My-ETS-19 | XP_021353499.1 | My-ETS-21 | XP_021353501.1 | Tandem duplication | Chr16 |
